# Innovative reconstruction protocol for complex alveolar ridge defects in posterior mandible: Case Report and technical analysis

**DOI:** 10.3389/fbioe.2025.1693842

**Published:** 2025-12-10

**Authors:** Jian Feng, Kewen Jia, Siyu Chen, Sicong Ren, Yuzhu Han, Mucong Li, Yidi Zhang, Yanmin Zhou

**Affiliations:** 1 Hospital of Stomatology, Jilin University, Changchun, China; 2 Jilin Provincial Engineering Laboratory of Intelligent Oral Treatment Technology, Changchun, China; 3 Shanghai Pudong New Area Eye and Dental Diseases Prevention and Treatment Center, Shanghai, China

**Keywords:** knife-edge ridge, *in situ* bone grafting, keratinized gingiva augmentation, open-suture technique, platelet-rich fibrin

## Abstract

**Background:**

Knife-edge ridges in posterior mandible present challenges for implant placement due to insufficient horizontal bone width and inadequate keratinized tissue, traditionally requiring secondary donor site surgery with associated morbidity.

**Case Description:**

A 46-year-old female with severe mandibular atrophy underwent two-stage reconstruction. First-stage surgery involved harvesting bone blocks from the knife-edge ridge, rotating them 180° counterclockwise, and securing for horizontal augmentation. After 7 months, second-stage surgery included implant placement with platelet-rich fibrin soft tissue augmentation using open-suture technique. Outcomes demonstrated horizontal bone gains of 4.3–4.8 mm, keratinized tissue increase of 2.9 mm, and absolute bone volume gain of 859.86 mm^3^. All three implants achieved successful osseointegration.

**Practical Implications:**

This novel staged protocol integrates *in situ* bone grafting with PRF-based soft tissue augmentation, specifically designed for posterior mandibular knife-edge ridges. By systematically combining these techniques, it eliminates donor site morbidity while achieving clinically significant tissue augmentation, offering practitioners a comprehensive autologous reconstruction approach that addresses the unique anatomical constraints and dual tissue deficiencies characteristic of this challenging region.

## 1 Background

Knife-edge alveolar ridges in the posterior mandible pose considerable challenges for implant rehabilitation (Shen et al., 2023). Although these ridges typically retain adequate vertical height following tooth loss, their insufficient horizontal dimension prevents stable implant placement without augmentation procedures (Thoma et al., 2018). The complexity increases substantially when keratinized tissue deficiency accompanies the bone defect (Chiapasco et al., 2020), requiring comprehensive reconstruction of both hard and soft tissues for predictable outcomes.

The posterior mandible presents unique anatomical constraints that limit reconstructive options (Schwarz et al., 2024). The proximity of the mental foramen and inferior alveolar nerve restricts both the extent of possible augmentation and available sites for local bone harvest (Schwarz et al., 2022). Although autogenous bone remains the gold standard for its osteogenic, osteoinductive, and osteoconductive properties (Zufia and Abella Sans, 2022), donor site morbidity represents a significant clinical limitation. Donor site complications occur in 27.8–63.6 percent of cases, with manifestations ranging from transient paresthesia to persistent pain, infection, and fracture (Wang et al., 2023).

Horizontal bone augmentation in the posterior mandible typically results in a mean gain of 4.8 mm using guided bone regeneration techniques. Autogenous bone blocks demonstrate superior volume stability compared to particulate grafts, maintaining 4.5 mm versus 3.7 mm of augmentation at 6 months (Benic et al., 2019; Smeets et al., 2022). Soft tissue reconstruction presents parallel challenges in achieving adequate peri-implant tissues. Adequate keratinized tissue width of at least 2 mm is essential for long-term peri-implant health (Koca-Unsal et al., 2022); however, palatal grafts required for soft tissue augmentation introduce additional surgical sites with associated morbidity (Ali et al., 2023). The development of platelet-rich fibrin (PRF) has provided promising alternative approaches for tissue regeneration without secondary donor site involvement. Published studies report enhanced bone defect fill rates with PRF in approximately 85 percent of cases when compared to conventional approaches (Miron et al., 2024; Lektemur Alpan and Torumtay Cin, 2020; Mijiritsky et al., 2021; Petrescu et al., 2021; Al-Maawi et al., 2021).

Various surgical approaches have been developed for posterior mandibular reconstruction. Rotation-based techniques, including pedicled segmental rotation (Khojasteh et al., 2019), rotational autotransplantation (Damlar et al., 2017), and island osteoperiosteal flaps (Jensen et al., 2010), employ 90° rotations for combined vertical-horizontal defects but require extensive osteotomies and rigid fixation of vertically positioned segments. Ridge splitting techniques (Holtzclaw et al., 2010; Pandey et al., 2022) achieve horizontal gains without donor sites but require minimum 2 mm crestal width—rarely present in severe knife-edge configurations. Neither approach systematically addresses concomitant keratinized tissue deficiency.

In the present case, we show a two-stage tissue regeneration protocol that we developed, which addresses both horizontal bone deficiency and keratinized tissue inadequacy through sequential interventions. The first stage involves harvesting bone blocks directly from the atrophic ridge, rotating them 180°, and repositioning for horizontal augmentation. The second stage combines implant placement with PRF-enhanced soft tissue augmentation using an open-suture technique that preserves periosteal vascularization. Then, at the end, the basic theory and clinical evidence of the technology are summarized to prove its feasibility.

This approach eliminates donor site morbidity while achieving substantial tissue gains through strategic timing and minimally invasive surgical techniques. The case report presents the clinical application and outcomes of this integrated protocol in a patient with severe posterior mandibular atrophy. While *in situ* bone grafting concepts have been described in the literature, the present approach differs fundamentally in three key aspects. First, we integrate a two-stage protocol that systematically addresses both horizontal bone deficiency and keratinized tissue inadequacy through a coordinated surgical sequence, rather than treating these deficiencies as separate clinical problems. Second, our technique specifically targets knife-edge ridges in the posterior mandible—an anatomically challenging region where traditional donor site options are severely limited by proximity to neurovascular structures. Third, we combine the rotated bone block technique with PRF-based soft tissue augmentation using an open-suture approach that eliminates the need for palatal grafts, achieving comprehensive autologous reconstruction without any secondary donor site morbidity.

## 2 Case presentation

### 2.1 Patient presentation and initial symptoms

This case report was prepared in accordance with the CARE guidelines and strictly adhered to the ethical principles outlined in the World Medical Association Declaration of Helsinki and were approved by the SJDKQ2024091 ethical committee.

A 46-year-old female presented seeking implant-retained restoration to restore masticatory function following prolonged edentulism in the mandibular left posterior region. The patient reported excellent general health without systemic conditions and denied tobacco use.

### 2.2 Clinical and radiological assessment

**FIGURE 1:**
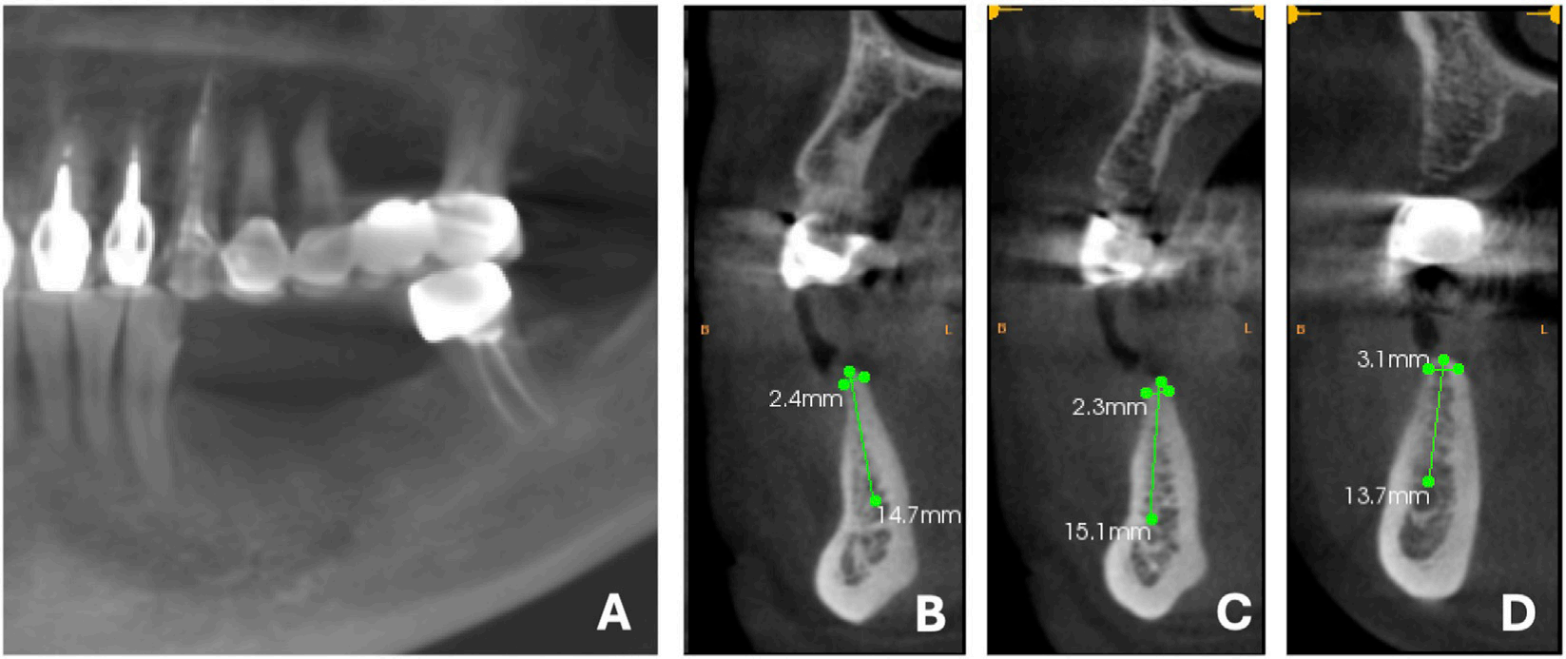
Preoperative imaging evaluation, **(A)** Surface tomography of the missing tooth site; **(B)** #34 Width and height of the remaining alveolar bone; **(C)** #35 Width and height of the remaining alveolar bone; **(D)** #36 Width and height of the remaining alveolar bone.

Cone-beam computed tomography (CBCT) revealed a characteristic knife-edge alveolar ridge (Cawood and Howell Classification IV) with severe horizontal bone deficiency. Measurements at 1 mm below the alveolar crest demonstrated only 2.3 mm width, though adequate vertical bone height remained (Figure 1). Clinical soft tissue examination revealed atrophic alveolar mucosa with insufficient keratinized gingival width, factors that would compromise long-term implant stability without intervention.

The patient expressed clear treatment objectives: restoration of masticatory function, minimized treatment duration, and reduced surgical trauma. Following comprehensive evaluation, we established a two-stage protocol: initial *in situ* external bone augmentation addressing horizontal deficiency, followed by implant placement with simultaneous PRF-based keratinized gingival augmentation. By dividing the procedure into two stages, we minimized the number of interventions while allowing for comprehensive autologous tissue reconstruction.

### 2.3 Integrating treatment planning and preoperative preparation

#### 2.3.1 Hard tissue augmentation design

The radiological analysis, along with a 1:1 3D printed model, was used to simulate the surgery for bone augmentation planning. This allowed for precise identification of bone removal locations and the positioning of bone blocks, ensuring the accuracy of the surgery through careful intraoperative measurements.

#### 2.3.2 Soft tissue augmentation design

PRF-assisted soft tissue augmentation surgery was performed 7 months after the bone augmentation surgery (Li et al., 2023). The keratinized gingival augmentation concurrently with implant placement using the open suture technique, achieving complete autologous soft tissue reconstruction. The open suture technique secured PRF membranes through strategic suture placement from the buccal mucoperiosteal surface, traversing the PRF membrane to the lingual flap tissue.

#### 2.3.3 Preoperative preparation

**FIGURE 2:**
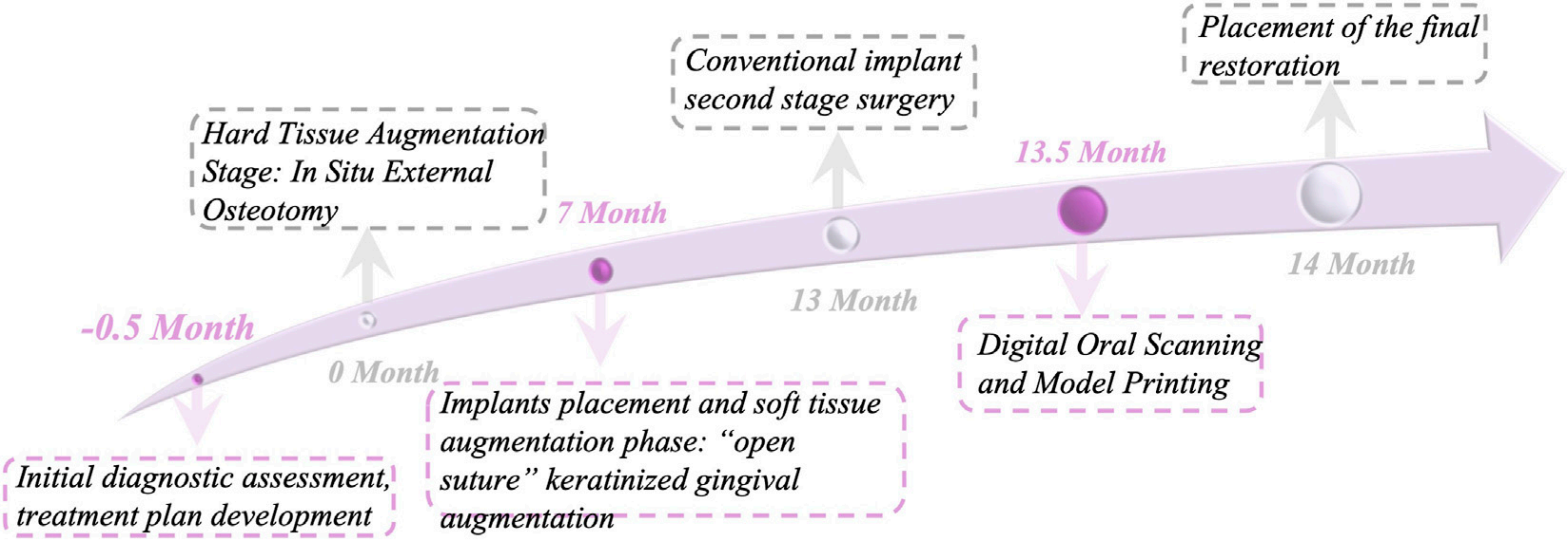
Overall surgical program design schedule.

Treatment planning discussions addressed all clinical and financial considerations before obtaining written informed consent (Figure 2). Antibiotic prophylaxis consisted of amoxicillin 500 mg administered orally 1 h preoperatively. The patient completed three 3-min oral rinses with 0.12% chlorhexidine solution. Before keratinized gingival augmentation, we collected 50 mL venous blood and processed it via centrifugation at 3,000 rpm for 10 min using 10 mL glass-coated tubes without anticoagulants.

### 2.4 Staged surgical implementation

#### 2.4.1 Integration process of surgical steps

After administering local anesthesia with articaine, the first stage of bone augmentation began. A crestal incision with lingual deviation was used, complemented by vertical releasing incisions on the proximal and distal buccal aspects. Full-thickness mucoperiosteal flap elevation achieved complete knife-edge ridge exposure. The mental foramen was identified under direct visualization and correlated with preoperative CBCT planning, ensuring adequate safety margins.

**FIGURE 3:**
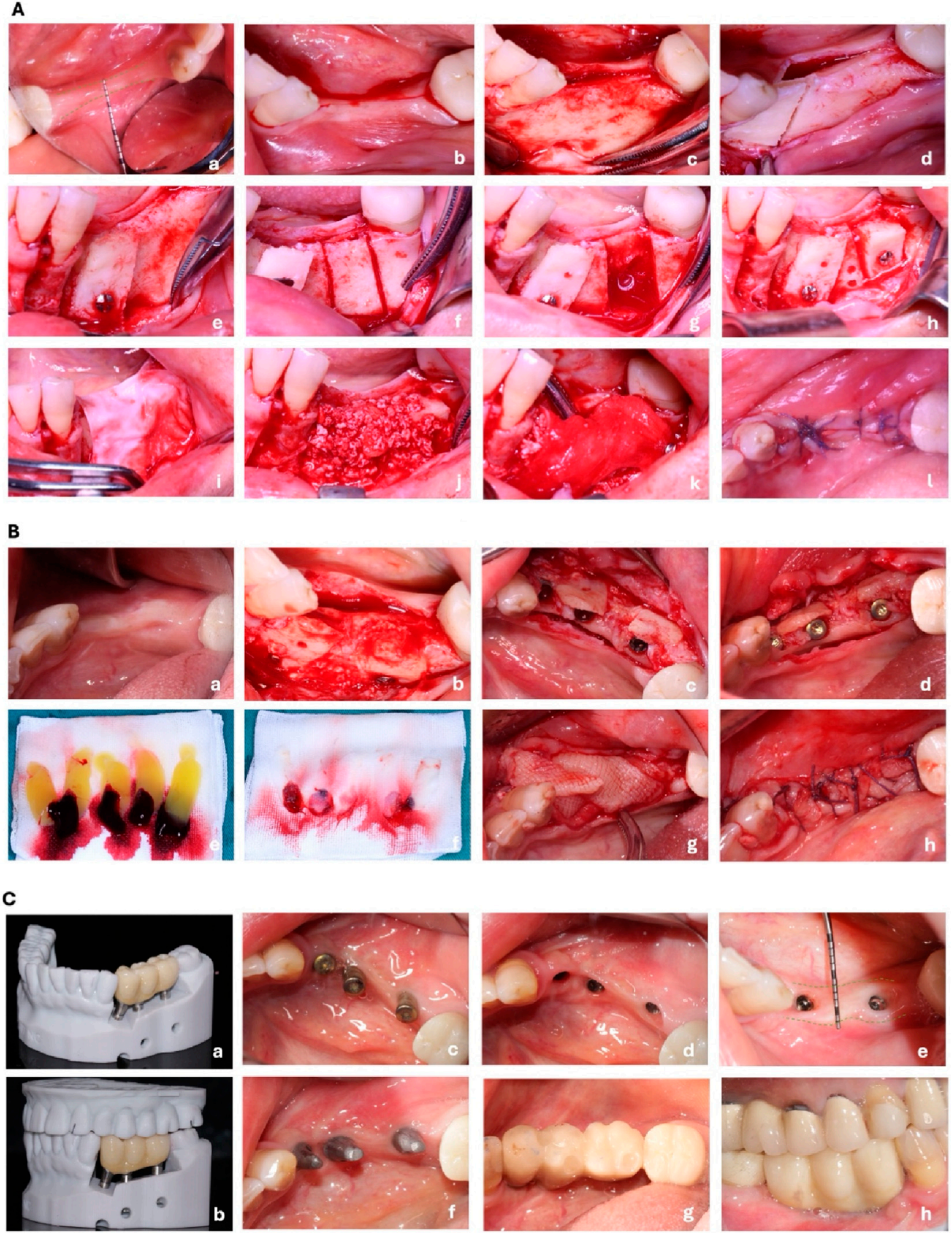
**(A)**: Surgical steps of the first stage - bone augmentation by *in situ* external method: **(a)** Intraoral view of the preoperative position of the missing teeth, and measurement of the remaining keratinized gingival width (2.8 mm) by periodontal probe; **(b)** Incision position; **(c)** Flap flap, exposing the position of the chin foramen; **(d)** Ultrasonic osteotome and proximal-medial extraction of the bone from the chin foramen; **(e)** Removal of the proximal-medial block of bone with a 180-degree counterclockwise rotation and fixation of the bone block with a microscrew titanium nail **(f)** Ultrasonic bone knife and distal bone extraction with chin foramen; **(g)** Bone condition in recipient/donor area; **(h)** Removal of distal bone block rotated 180° counterclockwise and fixed with micro-spiral titanium nails; **(I)** Bone augmentation area covered with resorbable collagen membrane; **(j)** Implantation of DBBM; **(k)** Periosteal nails to fix the resorbable collagen membrane; **(l)** Tension-free suture of the wound. **(B)**: Stage II - Implant Placement Surgery with “Open Suture” Keratinized Gingival Augmentation Surgical Procedure: **(a)** Preoperative intraoral view of the missing tooth position; **(b)** Incision, flap flap; **(c)** Implant cavity preparation and implant placement; **(d)** Installation of the overdenture screws; **(e,f)** Patient’s autologous PRF and compression to form a membrane; **(g)** Surgical area covered with multiple layers of PRF membrane; **(h)** Performing “Open Suture”. **(C)**: Prosthetic stage: **(a)** Mandibular digital model with the morphology of the final restoration; **(b)** Digital model of the maxillary and mandibular dentition; **(c)** Intraoral healing abutment; **(d)** Peri-implant cuff morphology; **(e)** Periodontal probe measurement of the keratinized gingival width (5.7 mm); **(f)** Placement of the permanent abutment; **(g)** Installation of permanent crowns occlusal surface morphology; **(h)** Occlusion of the final restoration.

Piezosurgical osteotomy allowed for the precise harvesting of bone blocks for dual block placement in the premolar (#34, #35) and molar (#36) regions. The osteotomy protocol comprised three sequential steps: horizontal corticotomy, vertical corticotomy, and bone block mobilization using double-edged chisels. Each bone block underwent 180° counterclockwise rotation, positioning the inferior thicker portion onto the superior narrow alveolar crest for horizontal augmentation. Titanium screws secured grafts at recipient-donor interfaces. A resorbable collagen membrane provided barrier function before primary wound closure (Figure 3A).

Second-stage surgery encompassed implant placement with concurrent soft tissue augmentation. Following local anesthesia and crestal flap elevation, we removed titanium screws and periosteal tacks. Sequential osteotomy preparation at sites #34-#36 preceded placement of ITI Straumann BLT SLActive 3.3 mm × 10 mm implants, achieving primary stability exceeding 35 Ncm. Cover screws were placed subsequently.

Five PRF blood clots were taken and pressed into membranes, which were then used to completely cover the wound before suturing. During the suturing process, we retained 1–2 mm thick keratinized mucosa on both sides to make it fit closely with the periosteum surface to provide keratinocytes. The PRF membrane, which was used as a soft tissue graft, was then sutured to the buccal and lingual flaps using interrupted sutures (Figure 3B).

#### 2.4.2 Optimized protocol for postoperative management

Our postoperative pharmacological regimen included antibiotics (amoxicillin 500 mg three times daily for 5 days), nonsteroidal anti-inflammatory drugs (ibuprofen 200 mg as needed), and 0.12% chlorhexidine oral rinses (twice daily for 2 weeks). Patients received instructions to avoid vigorous rinsing and mechanical cleansing of surgical sites for 2 weeks. Subsequently, gentle cleaning with soft-bristled toothbrushes was permitted while continuing to avoid hard foods.

## 3 Clinical outcome assessment

### 3.1 Radiographic evaluation of hard tissue augmentation

**FIGURE 4:**
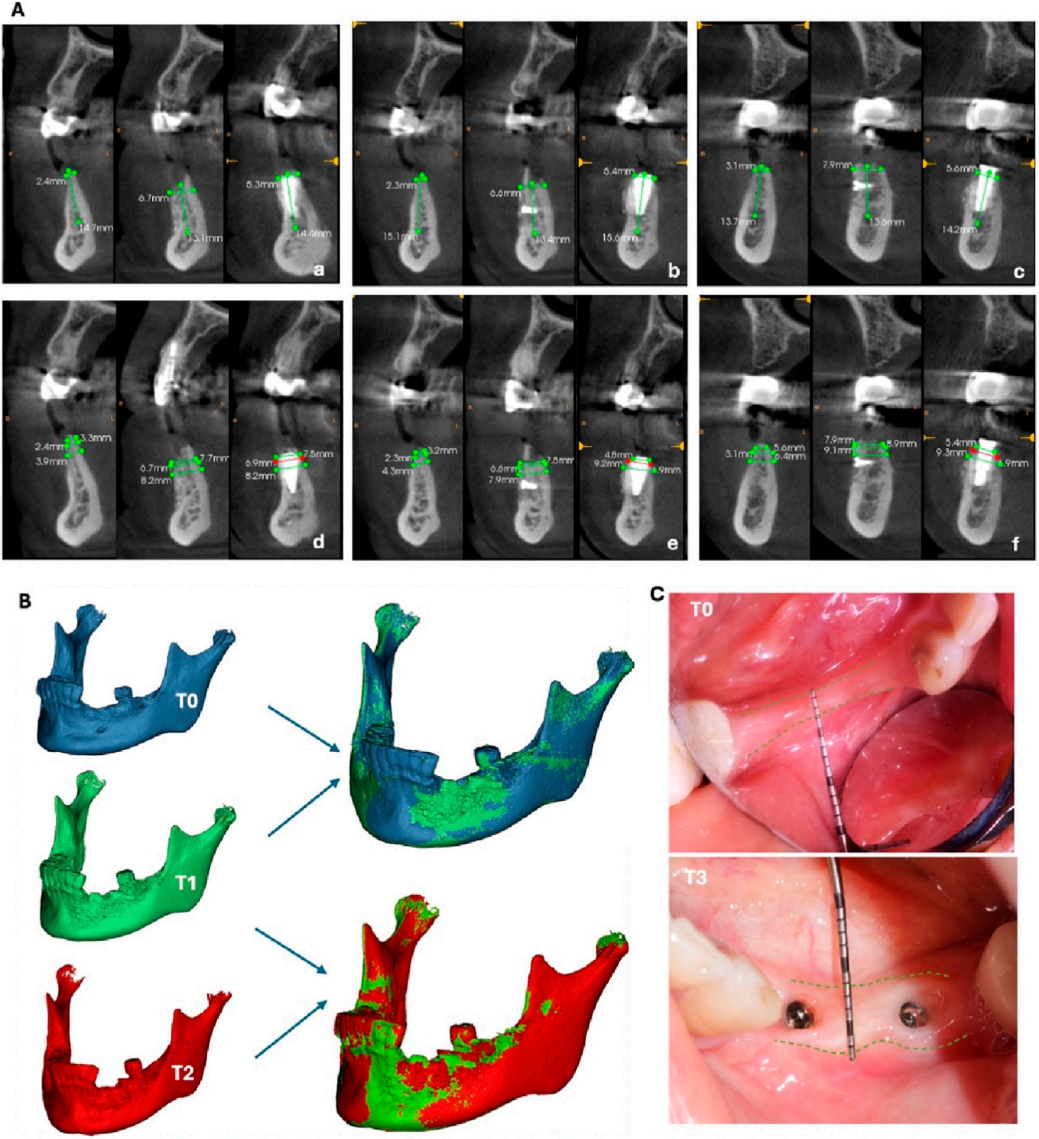
**(A)**: **(a)** Alveolar bone width and height of #34; **(b)** Alveolar bone width and height of #35; **(c)** Alveolar bone width and height of #36; **(d)** Alveolar bone width at 1, 2 and 3 mm below the top of the alveolar ridge/implant platform of #34, #35 **(e)** and #36 **(f)**. **(B,C)**: Perioperative bone volume changes and keratinized gingival augmentation following staged reconstruction. Data from standardized time points: T_0_ (preoperative), T_1_ (7 months post-bone grafting), T_2_ (6 months post-implant placement, equivalent to 13 months post-bone grafting), and T_3_ (final restoration stage, 14 months post-bone grafting). **(B)** bone volume changes; **(C)** keratinized gingival augmentation.

CBCT analysis was performed at various time points: T_0_ (preoperative), T_1_ (7 months post-bone grafting), T_2_ (6 months post-implant placement, equivalent to 13 months post-bone grafting), and T_3_ (final restoration stage, 14 months post-bone grafting), showing significant volumetric augmentation. (Figure 4A). Measurements at 1 mm below the alveolar crest revealed: site #34 increased from 2.4 mm to 6.7 mm (horizontal gain: 4.3 mm); site #35 from 2.3 mm to 6.6 mm (gain: 4.3 mm); and site #36 from 3.1 mm to 7.9 mm (gain: 4.8 mm) (Table 1). Radiographic assessment revealed complete graft incorporation without discernible interfaces. The DBBM particulate material interposed between bone blocks demonstrated optimal integration with autogenous bone.

**TABLE 1:** Semi-quantitative analysis of bone width and its changes at implant sites#34- #36. (BW: bone width measured at T_0_ (preoperative), T_1_ (7 months post-bone grafting), and T_2_ (6 months post-implant placement, 13 months post-bone grafting)).

Site	BW/Height	T_0_	T_1_	T_2_	T_1-0_	T_2-1_	T_2-0_
#34	1 mm	2.4	6.7	6.5	4.3	−0.2	4.1
	2 mm	3.3	7.7	7.9	4.4	0.2	4.6
	3 mm	4.9	8.2	8.2	3.3	0	3.3
#35	1 mm	2.3	6.6	4.8	4.3	−1.8	2.5
	2 mm	3.2	7.5	6.9	4.3	−0.6	3.7
	3 mm	4.3	7.9	9.2	3.6	1.3	4.9
#36	1 mm	3.1	7.9	5.4	4.8	−2.5	2.3
	2 mm	5.6	8.9	6.9	3.3	−2	1.3
	3 mm	6.4	9.1	9.3	2.7	0.2	2.9

Alveolar socket width (BW) at sites #34-#36 was measured at 1, 2, and 3 mm below the implant platform using CS 3D Imaging software for Bone width changes from baseline were calculated as T_1_-T_0_ and T_2_-T_0_, with interval changes between implant placement and final assessment designated as T_2_-T_1_. Some bone resorption occurred at 1 mm below the implant platform compared to immediate post-grafting measurements (Table 1).

The CBCT data at different time points were imported into Mimics Research 21.0 for three-dimensional reconstruction and image registration (Figure 4B). Boolean computational methods demonstrated an absolute bone volume increase of 859.86 mm^3^ at the surgical site 7 months (T1) following *in situ* external bone grafting.

### 3.2 Soft tissue augmentation

Three-month evaluation following second-stage PRF keratinized gingival augmentation revealed an increase in keratinized gingival width from 2.8 mm to 5.7 mm (Figure 4C)representing a gain of 2.9 mm.

### 3.3 Implant-related results

Second-stage surgery at T_1_ (7 months post-bone grafting) comprised implant placement with simultaneous PRF keratinized gingival augmentation. Stage II uncovering was performed at T_2_ (6 months post-implant placement, corresponding to 13 months post-bone grafting). Resonance frequency analysis revealed a mean implant stability quotient (ISQ) of 77, confirming successful osseointegration. Digital impressions were obtained 2 weeks following Stage II surgery, with definitive restoration delivery 2 weeks thereafter. This timeline demonstrates the efficiency of our staged management approach (Figure 3C).

At 6-month follow-up, all implants remained functional with 100% survival rate, fulfilling Albrektsson success criteria. No evidence of peri-implant inflammation was observed, validating the reliability and predictability of this technique for reconstructing knife-edge alveolar ridges in posterior mandibular regions.

### 3.4 Complication management and clinical management

Two months post-implant placement, clinical examination revealed localized bone exposure. We addressed this complication through precise contouring of exposed sharp osseous margins using an ultrasonic osteotome, carefully preserving augmented bone volume. This minimally invasive intervention successfully resolved the exposure while optimizing conditions for soft tissue healing.

## 4 Discussion

### 4.1 Technical innovation and clinical outcomes

The *in situ* external bone grafting technique represents a significant advancement in managing knife-edge alveolar ridges in the posterior mandible. By harvesting bone blocks directly from the atrophic ridge and rotating them 180° counterclockwise, we achieved horizontal bone gains of 4.3–4.8 mm while completely eliminating the morbidity typically associated with secondary donor sites. This outcome demonstrates clear advantages over conventional bone block grafting approaches, which typically yield 4.0–5.0 mm augmentation but carry a substantial donor site complication rate of 27.8%–63.6% (Wang et al., 2023). The volumetric analysis demonstrating 859.86 mm^3^ absolute bone volume increase at 7 months confirms the regenerative potential of this approach.

The biological success of this technique results from several interconnected factors. Specifically, the 180-degree rotation strategically positions the thicker inferior cortical portion of the knife-edge ridge toward the occlusal surface, thereby creating a substantially wider osseous platform. Simultaneously, this rotation transforms the anatomical configuration from a one-wall defect into a four-wall bone defect structure, which is known to provide superior biomechanical stability for bone regeneration.

The soft tissue outcomes achieved through the open-suture PRF technique proved equally significant. The 2.9 mm keratinized tissue gain exceeds the 2 mm threshold necessary for peri-implant health (Koca-Unsal et al., 2022) while eliminating the 14%–33% morbidity associated with palatal harvest required for traditional free gingival grafts (Ali et al., 2023). This outcome aligns with our previously reported results using the same technique (2.81 ± 1.12 mm), confirming reproducibility (Yuemeng et al., 2023). The preservation of periosteal blood supply through the open-suture design, without requiring flap repositioning, maintained the three-dimensional architecture of both hard and soft tissues while facilitating adequate keratinized tissue formation.

Our technique differs from existing approaches in several key aspects. Unlike 90° rotation methods that create vertically suspended segments requiring complex pedicle preservation and rigid fixation, our 180° horizontal rotation maintains simpler execution with inherent stability—rotated blocks rest on the knife-edge crest rather than being suspended vertically. Compared to ridge splitting techniques that require adequate crestal width and trabecular bone, our approach specifically targets knife-edge ridges too narrow for splitting. The technical advantages include: preserved dual vascularity through parent bone contact and intact periosteum, simplified fixation due to horizontal orientation, and integrated PRF-based soft tissue reconstruction achieving 2.9 mm keratinized tissue gain. The single complication (localized exposure) managed conservatively compares favorably to reported complications including segment necrosis in rotation techniques and buccal plate sequestration (0%–6.8%) in ridge splitting. Compared to mainstream approaches, our technique achieved horizontal gains of 4.3–4.8 mm, comparable to conventional autogenous block grafts from iliac crest, ramus, or symphysis which typically yield 4.0–5.0 mm, while eliminating the 27.8%–63.6% donor site complication rate associated with these procedures. Unlike tooth-root grafts that depend on availability of suitable extracted teeth, our approach utilizes the atrophic ridge itself. Contemporary GBR + PRF protocols typically employ bone substitutes and still require palatal grafts for adequate keratinized tissue reconstruction, introducing 14%–33% morbidity from palatal harvest. Our integrated approach distinctively achieves both hard tissue augmentation and soft tissue reconstruction (2.9 mm keratinized tissue gain) through fully autologous materials without any secondary donor site. The key clinical advantages of the present technique compared to conventional approaches are summarized in Table 2.

**TABLE 2:** Key clinical advantages of the present technique.

Clinical advantage	Present technique	Conventional approaches
Donor site	None	Required (bone:iliac/ramus/symphysis; soft tissue: Palate)
Donor site complications	0%	27.8%–63.6% (bone harvest) + 14–33% (palatal harvest)
Tissue reconstruction	Fully autologous (*in situ* bone + PRF)	Multiple donor sites or non-autologous materials
Horizontal bone gain	4.3–4.8 mm	4.0–5.0 mm
Keratinized tissue gain	2.9 mm	Requires separate palatal graft

### 4.2 Patient selection and long-term considerations

Establishing appropriate patient selection criteria remains essential for predictable outcomes. Based on this case and our accumulated clinical experience, we propose the following case selection criteria.

#### 4.2.1 Inclusion criteria

(1) Cawood and Howell Class IV knife-edge ridge in the posterior mandible; (2) adequate vertical bone height (≥6–7 mm from vital structures) permitting safe bone block harvest; (3) horizontal bone width at 1 mm below crest of 2–4 mm, allowing osteotomy without complete ridge perforation; (4) insufficient keratinized tissue width (<2 mm) requiring soft tissue augmentation; (5) absence of active infection or untreated periodontal disease; (6) systemically healthy patients or well-controlled medical conditions (HbA1c <7.0% for diabetic patients).

#### 4.2.2 Exclusion criteria

(1) Vertical bone deficiency (<6 mm from vital structures) where PSR techniques would be more appropriate; (2) adequate ridge width (>5 mm) suitable for direct implant placement or ridge splitting; (3) severe bone quality compromise (heavily irradiated bone, active bisphosphonate therapy); (4) uncontrolled systemic conditions affecting bone healing (uncontrolled diabetes, active chemotherapy, heavy smoking >20 cigarettes/day); (5) anatomical limitations including mental foramen position <3 mm from planned osteotomy site; (6) patient unwillingness to undergo staged procedures.

The six-month follow-up demonstrated successful osseointegration with ISQ values of 77% and 100% implant survival, yet longer-term evaluation remains necessary to establish definitive stability patterns. The observed bone resorption at 1 mm below the implant platform (0.2–2.5 mm) between post-grafting and post-implant measurements reflects expected remodeling rather than pathological loss.

### 4.3 Clinical implications and limitations

Although the staged nature of our protocol extends the overall treatment timeline to 13 months from initial surgery to final restoration, this extended duration reflects fundamental biological requirements rather than surgical convenience. The deliberate seven-month interval between initial bone grafting and implant placement permitted complete osseointegration of the augmented bone before subsequent soft tissue manipulation, likely contributing substantially to the favorable clinical outcomes observed in this case. This timeline compares favorably with alternative approaches requiring iliac crest harvest, which often necessitate hospitalization and extended recovery periods. The elimination of secondary donor sites represents the primary advantage, particularly relevant for posterior mandibular reconstruction where anatomical constraints limit harvest options and traditional approaches carry risks including inferior alveolar nerve injury and potential mandibular fracture (Wang et al., 2023).

The single complication of localized bone exposure at 2 months, successfully managed through conservative recontouring, suggests an acceptable safety profile when proper technique is employed. The complete reliance on autologous materials eliminates concerns regarding disease transmission, immunological reactions, and the substantial costs associated with commercial bone substitutes.

Several limitations should be acknowledged when interpreting these results. The single case presentation cannot establish broader applicability across diverse clinical scenarios, though we have successfully applied this technique in additional patients with similar defects awaiting comprehensive documentation. The absence of histomorphometric analysis limits understanding of bone quality and remodeling patterns within grafted sites. The lack of standardized patient-reported outcome measures prevents comprehensive assessment of treatment impact on quality of life.

## 5 Conclusion

This single case report demonstrates the potential of a staged reconstruction approach for managing knife-edge alveolar ridge defects through *in situ* external bone grafting combined with PRF-based soft tissue augmentation. In this case, the technique achieved substantial horizontal bone gains (4.3–4.8 mm) and adequate keratinized tissue width increase (2.9 mm) while eliminating donor site morbidity through exclusive use of autologous materials. However, as a single-case presentation, these findings have inherent limitations in generalizability. Prospective case series and controlled comparative studies are essential to establish the reproducibility, predictability, and definitive clinical guidelines for this approach before broader clinical application can be recommended. The favorable outcomes observed in this case suggest this technique may offer a viable alternative for managing complex posterior mandibular defects with reduced surgical morbidity, warranting further systematic investigation.

## Data Availability

The original contributions presented in the study are included in the article/supplementary material, further inquiries can be directed to the corresponding authors.
